# Investigation of Intestinal Microbiota and Fecal Calprotectin in Non-Toxigenic and Toxigenic *Clostridioides difficile* Colonization and Infection

**DOI:** 10.3390/microorganisms8060882

**Published:** 2020-06-11

**Authors:** Sung-Hee Han, Joowon Yi, Ji-Hoon Kim, Hee-Won Moon

**Affiliations:** 1 BioCore Co. Ltd., Biotechnology, Yongin 64844, Korea; hansungh@bio-core.com; 2 Samkwang Medical Laboratories, Seoul 06742, Korea; caod.alt@smlab.co.kr; 3 Advanced BioVision Inc., Incheon 21999, Korea; patriet80@gmail.com; 4 Department of Laboratory Medicine, Konkuk University School of Medicine, Seoul 05030, Korea

**Keywords:** *Clostridioides difficile* infection, colonization, microbiota, calprotectin

## Abstract

In this study, we aimed to evaluate the composition of the intestinal microbiota and level of fecal calprotectin in *Clostridioides difficile-*colonized patients. We included 102 *C. difficile* non-colonized (group I), 93 *C. difficile* colonized subjects (group II), and 89 diarrhea patients with *C. difficile* (group III). Chao1 index for alpha diversity and principal coordinate analysis was performed for beta diversity using QIIME. The mean relative abundance in each group was compared at the phylum and genus levels. Fecal calprotectin was measured using EliA calprotectin (Thermo Fisher Scientific). Group II showed significantly lower levels of *Sutterella*, *Blautia*, *Ruminococcus*, *Faecalibacterium*, *Bilophila*, and *Ruminococcaceae* and higher levels of *Enterobacteriaceae* compared to group I (*p* = 0.012, 0.003, 0.002, 0.001, 0.027, 0.022, and 0.036, respectively). Toxigenic *C. difficile* colonized subjects showed significantly lower levels of *Prevotella*, *Phascolarctobacterium*, *Succinivibrio*, *Blautia*, and higher levels of *Bacteroides*. The level of fecal calprotectin in group III was significantly higher than those in group I and group II (*p* < 0.001 for both). These data could be valuable in understanding *C. difficile* colonization process and the microbiota and inflammatory markers could be further studied to differentiate colonization from CDI.

## 1. Introduction

*Clostridioides difficile*, previously known as *Clostridium difficile*, is an anaerobic Gram-positive, spore-forming bacterium and a common cause of antibiotic-associated nosocomial diarrhea in the industrialized world [1,2]. After the use of antibiotics, microbiota-mediated colonization resistance is decreased partly by the reduced diversity of the intestinal microbiota [3,4,5,6,7]. Decreased diversity and alteration of the intestinal microbiota in *C. difficile* infection (CDI) has been shown in previous studies using various techniques from culture-based methods to high throughput sequencing [5,7,8,9,10,11]. The current standard diagnosis of CDI is made from the presence of toxigenic *C. difficile* in patients with significant diarrhea, defined as unexplained and new-onset (≥3 unformed stools in 24 h) [12]. Unfortunately, current diagnostic tests for CDI detect toxigenic *C. difficile* or its toxins or toxin gene. Many assays cannot differentiate between colonization and infection. This causes a false classification of *C. difficile-*colonized patients with diarrhea as CDI [13,14]. Meanwhile, direct fecal biomarkers related to intestinal inflammation, such as calprotectin, were shown to significantly increase in CDI and were proposed as severity markers [15,16]. Although recent guidelines do not recommend inflammatory markers for diagnosis or prognosis determination in CDI due to insufficient data, the research is ongoing and could be evaluated for differentiation between colonized patients and infection [13,17]. To the best of our knowledge, data for the human microbiota composition and inflammatory status in *C. difficile-*colonized subjects is sparse and there are no data on the comparison of toxigenic and non-toxigenic *C. difficile* colonized subjects.

The present study aimed to evaluate the composition of the intestinal microbiota and inflammatory status in *C. difficile* colonized subjects and compare them with those in non-colonized control and patients with significant diarrhea. We also compared the differences between toxigenic and non-toxigenic *C. difficile* colonized subjects.

## 2. Materials and Methods

### 2.1. Clinical Samples

This study was approved by the Institutional Review Board of the Konkuk University Medical Center, Seoul, Korea (a tertiary referral hospital). In addition to this, all methods were performed in accordance with the relevant guidelines and regulations. From March 2018 to June 2019, we screened for *C. difficile* colonization in 812 residual fecal samples from individuals who visited our center for a general health examination. Korean adults (>30 years old) were included and subjects with obesity (body mass index > 25), other ethnicity or history of recent hospitalization were excluded. *C. difficile* colonization was firstly assessed by glutamate dehydrogenase (GDH) testing using VIDAS *C. difficile* GDH on the VIDAS instrument (bioMérieux, Marcy-l’Etoile, France) according to the manufacturer’s instructions. Among the screened samples, 93 (11.4%) were GDH-positive. We defined “*C. difficile* colonization” as positive GDH in the absence of CDI symptoms [14]. We included randomly selected 102 GDH-negative samples (group I, *C. difficile* non-colonized) and 93 GDH-positive samples (group II, *C. difficile* colonized) for further study. Toxin status was determined by *tcdB* gene real-time PCR (Xpert *C. difficile* system, Cepheid, Sunnyvale, CA, USA). The Xpert *C. difficile* is an automated, real-time multiplex PCR assay, which detects the genes for *tcdB*, binary toxin (*cdt*), and a point mutation at position 117 associated with hypervirulent 027/NAP1/BI strains. A maximum valid Ct is 37 for *tcdB* and *cdt* according to the manufacturer’s instructions. Among the 93 *C. difficile-*colonized subjects (group II), 13 (14%) were *tcdB* gene positive. In addition, we collected 89 GDH-positive samples from hospitalized patients with significant diarrhea (≥3 unformed stools in 24 h) [3,18]. They received antibiotics and most patients were from the hematologic and gastrointestinal department. We excluded the patients with laxative use in 48 h before sample collection to rule out other reasons for diarrhea. 

They included 20 *tcdB* gene-negative and 69 *tcdB* gene-positive samples (group III, diarrhea with *C. difficile*). A total of 284 samples were analyzed and the characteristics of each group are summarized in Table 1. Upon reception, stool samples were stored at −70 °C until use. This study required neither study-specific nor any other interventions and the data were analyzed anonymously. Therefore, written informed consent from the enrolled patients was waived by the ethics committee.

### 2.2. Microbiome Analysis

DNA extraction from a stool sample was performed using a QIAamp DNA stool mini kit (Qiagen, Valencia, CA, USA) and bacterial 16S rRNA genes were amplified using an Ion 16S™ Metagenomics Kit (ThermoFisher Scientific, Waltham, MA, USA) according to the manufacturer’s protocol. The kit included 2 primer tubes with 3 primer sets that amplify the hypervariable regions of 16S rRNA (V2, V4, V8 and V3, V6–7, V9, respectively). After PCR amplicons were purified using Agencourt AMPure^®^ XP beads (Beckman Coulter, Indianapolis, IN, USA), sequencing libraries were prepared using an Ion Plus Fragment Library Kit and Ion Xpress™ Barcode Adapters (ThermoFisher Scientific, Waltham, MA, USA). Prepared libraries were quantified using a High Sensitivity DNA kit on an Agilent 2100 Bioanalyzer (Agilent Technologies, Santa Clara, CA, USA). Template preparation and sequencing were performed using the Ion Chef™ System and Ion S5™ XL system with Ion 530™ Chip Kit (ThermoFisher Scientific, Waltham, MA, USA). After filtering out low quality and polyclonal reads, and trimming any adaptor sequences at the 3′ end, the sequencing data were exported as FASTQ files and were processed using the Quantitative Insights into Microbial Ecology (QIIME) pipeline 1.9.1 [19]. After quality filtering, 26,634,236 sequences were obtained, with a mean of 89,165 sequences per sample (min: 16,585, max: 335,275). Operational taxonomic units (OTUs) were clustered based on 97% sequence similarity with at least 10 identical sequences and assigned against the curated Greengenes v.13.8 reference database at the QIIME web site (http://qiime.org/home_static/dataFiles.html).

Alpha and beta diversity measures were calculated by QIIME [20]. Alpha diversity assessment was based on observed OTUs, including unidentified OTUs, and Chao1 index. Microbial diversity was visualized using principal coordinate analysis (PCoA) and microbial diversity between samples was assessed by qualitative (unweighted UniFrac) and quantitative (weighted UniFrac) distances. The mean relative abundance (percentage among all reads) in each group was compared at the phylum and genus levels.

### 2.3. Calprotectin Measurement

After thawing, fecal samples were extracted using EliA Stool Extraction Kit (Thermo Fisher Scientific, Waltham, MA, USA). The extraction tubes were pre-filled with 750 μL of EliA Calprotectin Extraction Buffer. Fecal calprotectin was measured using EliA calprotectin on the Phadia 250 system (Thermo Fisher Scientific, Waltham, MA, USA). The wells of the EliA Calprotectin were coated with monoclonal antibodies against calprotectin, and the calprotectin levels were quantified by fluoroenzyme immunoassay. Phadia 250 measures calprotectin concentrations in ng/mL, and by using a conversion factor given by the lot-specific code of the EliA Calprotectin Well, the results were automatically converted to mg/kg.

### 2.4. Statistical Analysis

The difference between the continuous variables was analyzed using Student’s *t*-test or the Mann–Whitney U test, and that between categorical variables was analyzed using the chi-squared test, Fisher’s exact test, or the McNemar test. The Kruskal–Wallis test and one-way analysis of variance (ANOVA) were used to assess the differences between groups. Permutational multivariate analysis of variance (PERMANOVA) between groups was performed using QIIME. Statistical analysis was performed using MedCalc Statistical Software (v. 15.8, MedCalc Software, Mariakerke, Belgium) and IBM SPSS Statistics 22.0 (IBM Corporation, Armonk, NY, USA). *p* values less than 0.05 were considered statistically significant.

## 3. Results

### 3.1. Comparison of Alpha Diversity of the Intestinal Microbiota Across Different Groups

**Figure 1:**
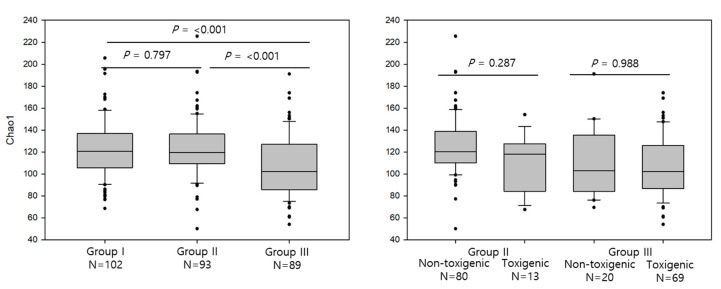
Alpha diversity (Chao1 index) in group I (*C. difficile* non-colonized), group II (*C. difficile* colonized), and group III (diarrhea with *C. difficile*) are shown in left and the comparisons between non-toxigenic and toxigenic *C. difficile* within group II and group III are shown in right. Each *p*-value (I vs. III, I vs. II, II vs. III, and non-toxigenic vs. toxigenic *C. difficile*) was presented. The box plot shows the median and interquartile values. The whiskers indicate 1.5 times the interquartile range above and below the 75th and 25th percentiles. The circles indicate the outliers.

We evaluated the differences in alpha diversity between group I (*C. difficile-*non colonized), group II (*C. difficile-*colonized), and group III (diarrhea with *C. difficile*). The distribution of the Chao1 indexes in each group is presented in Figure 1. The median Chao1 index of group III [median, interquartile range (IQR), 102.3, 85.7–127.1] was significantly lower than that of group I and group II (120.8, 105.7–136.9 and 119.5, 109.2–136.3, *p* < 0.001 for both). The median Chao1 index between group I and group II was not significantly different (*p* = 0.797). The median Chao1 index between subjects with non-toxigenic and toxigenic *C. difficile* was not significantly different in group II and III (*p* = 0.287 and 0.988, respectively).

### 3.2. Comparison of Beta Diversity of the Intestinal Microbiota Across Different Groups

**Figure 2:**
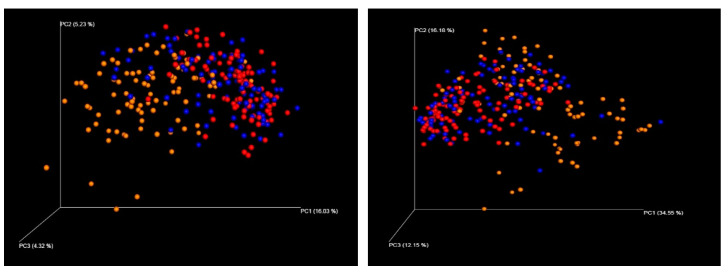
Evaluation of beta diversity in group I (red), II (blue), and III (orange). Principal coordinate analysis (PCoA) was performed using unweighted (left) and weighted (right) UniFrac distances of 16S rRNA gene sequences. Each axis represents inter-sample variation.

PCoA using unweighted and weighted UniFrac matrix was performed to evaluate the beta diversity between each group. In unweighted UniFrac analysis, group I vs. III and group II vs. III clustered separately (both *p* = 0.001 by PERMANOVA), while group I and II could not be separated (*p* = 0.057 by PERMANOVA). In weighted UniFrac analysis, all three groups clustered separately (*p* = 0.017, 0.001, and 0.001 for group I vs. II, group I vs. III, and group II vs. III, respectively) (Figure 2). In group II, subjects with non-toxigenic and toxigenic *C. difficile* clustered separately both in unweighted and weighted UniFrac matrix (both *p* = 0.001). In group III, subjects with non-toxigenic and toxigenic *C. difficile* clustered separately only in unweighted UniFrac matrix (*p* = 0.001 in unweighted and 0.721 in weighted UniFrac matrix).

### 3.3. Comparison of Mean Relative Abundance in Each Group at the Phylum Level

The comparison of mean relative abundance in groups I, II, and III at the phylum level is shown in Table 2. Group III showed significantly lower levels of *Bacteroidetes* (*p* < 0.001 and 0.001 compared with groups I and II, respectively) and significantly higher levels of *Proteobacteria* compared to group I (*p* < 0.001). Comparing subjects with non-toxigenic and toxigenic strains in group II, those with toxigenic *C. difficile* colonized subjects showed significantly lower levels of *Firmicutes* (*p* < 0.001). In group III, there was no significant difference in composition between patients infected with non-toxigenic and toxigenic *C. difficile* strains (Table 3).

### 3.4. Comparison of Mean Relative Abundance in Each Group at the Genus Level

The comparison of mean relative abundance in groups I, II, and III at the genus level is shown in Table 4. Group III showed a significantly lower mean relative abundance of many genera (*Prevotella*, *Lachnospira*, *Blautia*, *Ruminococcus*, *Phascolarctobacterium*, *Faecalibacterium*, *Bilophila*, unassigned genus of *Ruminococcaceae*, and *Lachnospiraceae*) compared with those in groups I and II. Group III also showed *a* significantly higher mean relative abundance of *Parabacteroides*, *Serratia*, *Veillonella*, *Enterococcus*, and an unassigned genus of *Enterobacteriaceae.* Group II showed a significantly lower abundance of *Sutterella*, *Blautia*, *Ruminococcus*, *Faecalibacterium*, *Bilophila*, and an unassigned genus of *Ruminococcaceae* and higher abundance of an unassigned genus of *Enterobacteriaceae* compared with those in group I (*p* = 0.012, 0.003, 0.002, 0.001, 0.027, 0.022, and 0.036, respectively). Moreover, toxigenic *C. difficile* colonized subjects showed a significantly lower abundance of *Prevotella*, *Phascolarctobacterium*, *Succinivibrio*, *Blautia*, and an unassigned genus of *Ruminococcaceae*, and higher abundance of *Bacteroides* and an unassigned genus of *Enterobacteriaceae* compared with non-toxigenic *C. difficile* colonized subjects (*p* = 0.014, <0.001, 0.019, 0.006, <0.001, 0.035, and 0.044, respectively). In group III, there was no significant difference between patients with non-toxigenic and toxigenic *C. difficile* (Table 5).

### 3.5. Comparison of Calprotectin Levels between Groups

**Figure 3:**
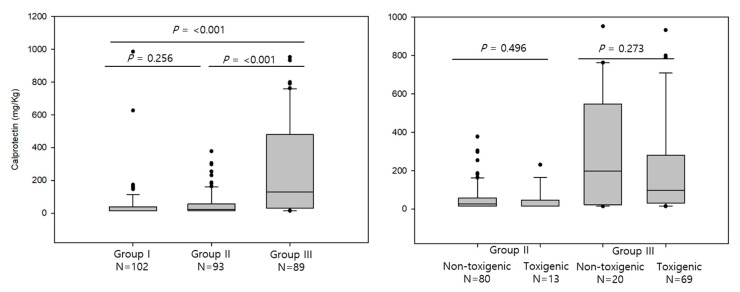
Fecal calprotectin levels in group I (*C. difficile* non-colonized), group II (*C. difficile* colonized), and group III (diarrhea with *C. difficile*) are shown in left and the comparisons between non-toxigenic and toxigenic *C. difficile* within group II and group III are shown in right. Each *p*-value (I vs. III, I vs. II, II vs. III and non-toxigenic vs. toxigenic *C. difficile*) was presented. The box plot shows the median and interquartile values. The whiskers indicate 1.5 times the interquartile range above and below the 75th and 25th percentiles. The circles indicate the outliers.

We compared fecal calprotectin levels in groups I, II, and III and its distribution is presented in Figure 3. The fecal calprotectin level in group III (median and IQR, 199.0 and 34.3–658.3 mg/kg) was significantly higher than those in group I and group II (15.0, 15.0–39.5 and 23.0, 15.0–57.0 mg/kg, respectively, *p* < 0.001 for both). There was no significant difference between group I and group II (*p* = 0.256). The fecal calprotectin levels were not significantly different between individuals with non-toxigenic and toxigenic *C. difficile* in both group II and group III (*p* = 0.496 and 0.273, respectively).

## 4. Discussion

The asymptomatic colonization rate of toxigenic *C. difficile* among healthy individuals ranges from 1.8 to 15% [14,21,22]. However, most studies were based on point prevalence detection and methodology and the target population was variable. In our study, *C. difficile* colonization among the screened population were 11.4% and the toxigenic portion among colonized *C. difficile* was relatively low (14%), which was lower than our previous studies performed in the same institution using the same method [23]. This difference might be due to different populations and different criteria in this study, which only included adult, non-obese individuals among visitors for a general health examination. The toxigenic proportion was much higher in hospitalized patients with diarrhea [23].

As expected, the alpha diversity index (Chao1), as shown in Figure 1, was significantly lower in group III (diarrhea with *C. difficile*) compared with those in group I (*C. difficile* non-colonized) and group II (*C. difficile* colonized). Previous studies also showed decreased alpha diversity in CDI or antibiotic exposed group compared with the control [8,24]. As decreased species diversity could occur in many hospitalized patients without CDI, this feature might not be specific for CDI and could be a common feature in hospitalized patients with antibiotic exposure. The development of overt CDI needs additional host factors, such as immunity, age, or hospital stay [3,4,25]. In this study, the alpha diversity index between group I and group II did not significantly differ. Thus, the *C. difficile* colonization itself in healthy individuals does not seem to affect species diversity. The present study also showed that the alpha diversity in group II and group III was not affected by toxigenic status.

In PCoA analysis, group III clustered separately from group I and II by unweighted and weighted UniFrac analysis, which was consistent with previous studies [8,24]. This indicates that hospitalized patients with diarrhea have different beta diversity to healthy individuals. We could find a significant difference between group I and II by weighed UniFrac analysis. Thus, although the total species diversity did not change in *C. difficile* colonization (based on alpha diversity), the composition and abundance of microbiota were changed in *C. difficile* colonization. Regarding toxin status, subjects with non-toxigenic and toxigenic *C. difficile* clustered separately both in unweighted and weighted UniFrac matrix, indicating that the intestinal microbiota is further changed distinctly upon colonization of toxigenic *C. difficile* strains compared to non-toxigenic strains. In patients of group III, non-toxigenic and toxigenic *C. difficile* also clustered separately, but significant difference was observed only in the unweighted UniFrac matrix. Disruption of intestinal microbiota composition occurs in many hospitalized patients, which could weaken the difference between those colonized with non-toxigenic and toxigenic strains.

Our study showed different patterns of the composition of fecal microbiota in patients with diarrhea, as shown previously [8,26]. Specifically, Group III patients showed lower and higher abundance of *Bacteroidetes* and *Proteobacteria*, respectively, at the phylum level, and lower relative abundance of many genera, including butyrate-producing organisms and health-promoting bacteria [9,27]. Increased *Parabacteroides* or *Enterococcus*, which reflect a blooming phenomenon by reduced ecological niche competition [7,8,28,29], were also observed in group III which included various hospitalized patients. These features were noted similarly in non-toxigenic and toxigenic *C. difficile*, suggesting again that this disruption of microbiota was not specific to patients with toxigenic *C. difficile*.

There is very limited data on the composition of intestinal microbiota in *C. difficile* colonization and a few studies only described a very limited number of subjects [8,14,30,31]. In this study, we could find several distinct features in *C. difficile* colonization, including those due to the toxigenic status of the strains. Group II (*C. difficile* colonized) did not show significant differences compared with non-colonized subjects (group I) at the phylum level and the results at phylum levels were the sum of the changes of many genera. At the genus level, group II showed a significantly lower abundance of many genera and a higher abundance of *Enterobacteriaceae* compared to group I. This pattern of change was partly similar to that observed in CDI or hospitalized patients, indicating that the genus compositions of *C. difficile* colonized subjects lies between non-colonized and hospitalized patients. Moreover, toxigenic *C. difficile-*colonized subjects showed significant changes, such as a lower abundance of *Prevotella, Phascolarctobacterium, Succinivibrio, Blautia,* and an unassigned genus of *Ruminococcaceae* and higher abundance of *Bacteroides* and an unassigned genus of *Enterobacteriaceae* than non-toxigenic *C. difficile-*colonized subjects. These features were closer to the patterns observed in hospitalized patients with diarrhea. We could not find a significant difference in hospitalized patients with diarrhea based on the presence or absence of toxigenic *C. difficile*.

Fecal calprotectin is a 36.5 kDa molecule derived from the cytoplasm of neutrophils, mononuclear cells, and squamous epithelial cells and reflects the inflammatory status of the intestine [32]. The proposed clinical utility of this marker has been focused on inflammatory bowel disease (IBD) [33]. In CDI, fecal calprotectin has been shown to reflect severity [15,16,34]; however, this marker showed a non-specific increase in various inflammatory conditions of the intestines, thereby limiting its diagnostic utility [35,36]. In this study, we showed that *C. difficile-*colonized subjects, regardless of toxin status, had comparable fecal calprotectin levels to those of non-colonized subjects and significantly lower levels than in diarrhea patients with *C. difficile.* The *C. difficile-*colonized subjects seemed to show a non-inflammatory status, although they showed changes in the composition of intestinal microbiota (beta diversity). The diarrhea patients had significantly higher fecal calprotectin levels, reflecting in high inflammatory status, and the toxin status did not show significant differences in calprotectin levels, indicating the non-specific increase of this marker, as suggested by previous studies [34,35,36].

This study had several limitations. The cause-effect relationship between specific alterations of the microbiota and clinical status or colonization could not be elucidated in this study design. Many covariates such as age or food intake habit could also affect the intestinal microbiota composition [37]. The median age of group III was significantly higher than the other groups and old age itself could result in decreased diversity and change composition of gut microbiota in group III [7,37]. The old aged subjects were rare in general health examination and enrollment of elderly subjects was difficult. Several studies recently showed data regarding differences in the gut microbiota between sexes [38,39]. In this study, we could not evaluate data based on sex differences and could be further studied. In addition, the results of the OTU assignment could be different between algorithms or programs used for OTU analysis [37,40]. 

## 5. Conclusions

There were significant alterations in the alpha and beta diversity in intestinal microbiota in hospitalized patients with diarrhea, but these alterations were not significantly affected by the toxin status of *C. difficile.* We found significant differences in the composition of intestinal microbiota in apparently healthy *C. difficile* colonized subjects in comparison to *C. difficile* non-colonized subjects. Despite the significant difference in *C. difficile* colonization such as lower levels of *Sutterella*, *Blautia*, *Ruminococcus*, *Faecalibacterium*, *Bilophila*, and *Ruminococcaceae* and higher levels of *Enterobacteriaceae*, inflammatory status and alpha diversity were not significantly different between *C. difficile* colonized and non-colonized subjects. Further investigation of the intestinal microbiota and immunological studies could provide some insight into the conditions that allow for colonization and protect against disease progression.

## Figures and Tables

**Table 1:** Demographic information for the subject in each group.

Groups	Source	Number	Sex (M/F)	Age, Years (Median, IQR)
Group I	General examination	102	52/50	57.0, 46.8–63.3
*C. difficile-* non colonized				
Group II	General examination	93	61/32	54.0, 47.0–63.0
*C. difficile-*colonized				
Group III	Hospitalized patients	89	49/40	67.0, 49–79 *
Diarrhea with *C. difficile*				

IQR, interquartile values. * *p* = 0.001 and < 0.001 compared group I and group II, respectively.

**Table 2:** Comparison of the mean relative abundance (%) at phylum level in *C. difficile-* non colonized (group I), colonized (group II), and diarrhea with *C. difficile* (group III).

Phylum	Group I	Group II	Group III	*p*		
	(*n* = 102)	(*n* = 93)	(*n* = 89)	I vs. II	I vs. III	II vs. III
*Bacteroidetes*	43.85	43.22	32.41	0.911	<0.001	0.001
*Firmicutes*	33.55	29.25	32.45	0.065	0.924	0.542
*Proteobacteria*	20.91	25.75	32.67	0.096	<0.001	0.077
*Actinobacteria*	0.98	0.81	1.1	0.564	0.952	0.75
*Fusobacteria*	0.64	0.89	0.97	0.753	0.724	0.987

Phyla with mean relative abundance >1.0% were described in Table 2.

**Table 3:** Comparison of the mean relative abundance (%) of the composition of phyla between non-toxigenic and toxigenic *C. difficile* in group II and group III.

*Phylum*	Group II		*p*	Group III		*p*
	Non-Toxigenic	Toxigenic		Non-Toxigenic	Toxigenic	
	(*n* = 80)	(*n* = 13)		(*n* = 20)	(*n* = 69)	
*Bacteroidetes*	42.85	45.46	0.491	32.21	32.47	0.968
*Firmicutes*	31.15	17.57	<0.001	31.62	32.69	0.865
*Proteobacteria*	24.63	32.67	0.137	34.73	32.08	0.668
*Acinetobacter*	0.87	0.44	0.151	0.48	1.28	0.371
*Fusobacteria*	0.42	3.83	0.059	0.62	1.08	0.624

Phyla with mean relative abundance >1.0% were described.

**Table 4:** Comparison of the mean relative abundance (%) at genus level *C. difficile-*non-colonized (group I), colonized (group II), and diarrhea with *C. difficile* (group III).

*Genus*	Group I	Group II	Group III	*p*		
	(*n* = 102)	(*n* = 93)	(*n* = 89)	I vs. II	I vs. III	II vs. III
*Bacteroides*	26.68	26.44	23.48	0.993	0.448	0.543
Enterobacteriaceae *	11.42	16.59	22.1	0.036	<0.001	0.067
*Prevotella*	10.09	8.94	1.36	0.756	<0.001	<0.001
Ruminococcaceae *	8.08	6.13	1.68	0.022	<0.001	<0.001
Lachnospiraceae *	7.16	6.06	3.49	0.118	<0.001	0.001
*Sutterella*	3.13	1.97	1.38	0.012	0.002	0.441
Rikenellaceae *	1.95	1.58	1.06	0.35	0.013	0.215
*Lachnospira*	1.84	1.36	0.27	0.055	<0.001	<0.001
*Blautia*	1.62	1.15	0.5	0.003	<0.001	<0.001
*Ruminococcus*	1.61	0.79	0.14	0.002	<0.001	<0.001
*Phascolarctobacterium*	1.54	1.76	0.45	0.87	<0.001	0.008
*Coprococcus*	1.25	1.05	0.2	0.514	<0.001	0.514
*Parabacteroides*	1.42	1.92	4.23	0.215	0.001	0.011
*Serratia*	1.34	1.5	3.79	0.842	<0.001	<0.001
*Faecalibacterium*	1.25	0.88	0.38	0.001	<0.001	0.002
*Oscillospira*	1.08	0.98	1.41	0.574	0.562	0.379
*Bilophila*	1.08	0.69	0.3	0.027	<0.001	0.024
*Veillonella*	0.3	0.45	2.25	0.51	0.003	0.007
*Succinivibrio*	0.3	1.12	0	0.248	0.341	0.05
*Lactobacillus*	0.16	0.46	1.41	0.33	0.113	0.185
*Clostridium*	0.11	0.13	1.34	0.835	0.264	0.276
*Eubacterium*	0.08	0.21	1.41	0.822	0.291	0.239
*Enterococcus*	0.03	0.46	9.13	0.542	<0.001	<0.001

Only genera with mean relative abundance >1% were described in Table 4. * unspecified to a specific genus within this family.

**Table 5:** Comparison of the mean relative abundance (%) of the composition of genera between non-toxigenic and toxigenic *C. difficile* in groups II and III.

*Genus*	Group II		*p*	Group III		*p*
	Non-Toxigenic	Toxigenic		Non-Toxigenic	Toxigenic	
	(*n* = 80)	(*n* = 13)		(*n* = 20)	(*n* = 69)	
*Bacteroides*	25.03	35.11	0.035	19.23	24.71	0.312
Enterobacteriaceae *	15.26	24.75	0.044	25.77	21.03	0.368
*Prevotella*	9.83	3.47	0.014	0.7	1.55	0.445
Ruminococcaceae *	6.85	1.73	<0.001	2.62	1.4	0.22
Lachnospiraceae *	6.36	4.24	0.073	2.65	3.73	0.423
*Sutterella*	2.1	1.19	0.05	0.74	1.56	0.407
*Phascolarctobacterium*	2.02	0.21	<0.001	0.54	0.46	0.713
Rikenellaceae *	1.67	0.98	0.204	1.37	0.97	0.501
*Parabacteroides*	1.66	3.52	0.265	6.93	3.45	0.166
*Lachnospira*	1.43	0.94	0.25	0.49	0.2	0.395
*Serratia*	1.41	2.08	0.32	3.67	3.81	0.902
*Succinivibrio*	1.3	0	0.019	0	0	0.33
*Ruminococcus*	1.3	1.59	0.127	0.16	0.14	0.753
*Blautia*	1.26	0.45	0.006	0.3	0.56	0.297
*Coprococcus*	1.12	0.63	0.199	0.12	0.22	0.462
*Oscillospira*	0.91	1.42	0.158	1.53	1.38	0.84
*Pseudomonas*	0.43	1.57	0.161	0.71	0.6	0.865
*Fusobacteriim*	0.4	3.66	0.062	0.56	0.82	0.702
*Veillonella*	0.35	1.1	0.102	0.39	2.79	0.002
*Enterococcus*	0.5	0.19	0.793	15.23	7.36	0.278
*Acinetobacter*	0.3	0.04	0.662	1.17	0.21	0.306
*Lactobacillus*	0.52	0.05	0.051	0.13	3.54	0.043
*Clostridium*	0.12	0.16	0.636	0.05	1.71	0.378

* unspecified to a specific genus within this family.
